# Influence of the GABA Receptor Subunit Gene Polymorphism and Childhood Sexual Abuse on Processing Speed in Major Depression and Suicide Attempt

**DOI:** 10.3389/fpsyt.2021.712231

**Published:** 2021-10-18

**Authors:** Honglei Yin, Jia Guo, Qianqian Xin, Shuqiong Zheng, Xiang Xue, Enze Li, Ting Liu, Na Yan, John Keilp, J. John Mann

**Affiliations:** ^1^Department of Psychiatry, Nanfang Hospital, Southern Medical University, Guangzhou, China; ^2^Guangdong-Hong Kong-Macao Greater Bay Area Center for Brain Science and Brain-Inspired Intelligence, Guangdong, China; ^3^Department of Biostatistics, Columbia University, New York, NY, United States; ^4^Department of Psychiatry, Columbia University, New York, NY, United States; ^5^Division of Molecular Imaging and Neuropathology, New York State Psychiatric Institute, New York, NY, United States

**Keywords:** processing speed, polymorphism, childhood sexual abuse, suicide attempt, depression

## Abstract

**Background:** Suicide is moderately heritable and also more common in those who report childhood abuse. Previously, it was found that allele A of GABRG2 (GABA A receptor subunit gamma2) polymorphism rs211034 was protective in a suicide attempt (SA). Hence, it was proposed that rs211034 may interact with childhood trauma to influence cognitive deficits related to SA or depression risk. Genetic variants may predict the benefits of certain cognitive treatments.

**Methods:** A total of 52 individuals who had attempted suicide, 59 individuals with major depressive disorder (MDD) or bipolar depression who had not previously attempted suicide, and 90 healthy volunteers were subjected to the modified Suicide Stroop task and were clinically assessed using the Childhood Trauma Questionnaire (CTQ) and Hamilton Depression Scale-24 items (HAMD-24). rs211034 was genotyped using Sanger sequencing.

**Results:** After correcting for covariates, depressed participants displayed longer reaction times for all emotional conditions, including suicide-related words, compared with healthy controls. Depressed suicide attempters displayed longer reaction times for negative words than depressed non-attempters. Depressed non-attempters displayed higher interference scores for negative words compared with healthy controls. There was an interaction between rs211034 risk allele and the effects of reported childhood sexual abuse (CSA) on reaction time for all emotional words and suicide-related words. Carriers of the rs211034 risk allele A exhibited shorter reaction times, but the protective effects of this allele were eliminated in those exposed to reported CSA.

**Conclusion:** Only limited results were found regarding effects of a past suicide attempt on response times to emotional and suicide-related words, but there was an overall effect of major depression on slower response time. Protective genetic effects of the rs211034 A allele on this slowing were eliminated in those with a history of sexual abuse during childhood. Further research is needed to better characterize the mechanisms underlying the effects of childhood trauma on these genetic effects.

## Introduction

Despite major advances in available options to treat mental health problems over recent decades, suicide rates remain high (1). There are an estimated 800,000 suicides per year worldwide and 20–40 times more non-fatal suicide attempts (World Health Organization). At least in part, this discrepancy highlights the need to develop a better understanding of factors that contribute to risk for suicide (2). To achieve this, we need more and stronger research on the mechanisms of suicidal behavior.

Suicidal individuals are characterized by “cognitive rigidity” (3). Cognitive deficits have been proposed as candidate “endophenotypes” for research on the genetics of suicide (4). In MDD with and without suicidal behavior, cognitive deficits have been observed in the following domains, attention, executive function, memory and processing speed (5).

A paradigm often used to study executive function and processing speed in depression and/or suicidal behavior is the Emotional Stroop Task (EST), especially a variant known as the Suicide Stroop task (SST), which was adapted from EST. EST modifies the classic color-word Stroop task by replacing color-words with neutral and emotionally valenced stimuli, and it measures the degree to which emotionally valenced stimuli interfere with the effortful process of responding to a stimulus providing incongruous or conflicting information (6). SST can measure reaction time (RT) to identify the color of suicide-related words. This measurement can record the response times (latencies) for participants to identify the color of different words presented on the computer screen. Larger response latencies means greater attention interference due to the content of the presented words and lower processing speed (7).

Previous studies found that, increased response latencies on SST reflect attentional interference caused by the salience of suicide-related information (8), and SST can strengthen assessment of suicide risk and provide better understanding of cognitive impairments associated with suicidal behavior (9). To date, however, studies investigating the performance of SST in suicide report mixed results. Some find that the suicide attempters showed attentional bias toward suicide-related words, and this bias can predict a suicide attempt over the next 6 months (10). Other studies reported negative results (11, 12).

The Stroop effect has been mostly studied with languages combinated with alphabetic letters (e.g., English, etc.). Although Chinese characters are different from these alphabetic words, there was no significant difference in Stroop effect between Chinese and English characters (13). A study using Chinese EST verified the cognitive deficit in depression (14). But so far, there is no article using Chinese SST to explore the cognitive impairment of suicidal behavior.

Reported childhood trauma (CT) may be another important factor which can impact the occurrence and extent of cognitive deficits in depression and suicide. CT was associated with reduced attention for angry and sad facial expressions in a depressed sample (15), and it was also related with differentiation in brain responses to positive compared with negative faces (16). RTs in EST were best predicted by self-reported experiences of CT (17). Greater CT exposure was associated with slower processing speed in depressed subjects (18). CT was associated with increased risk of suicidal behavior, and adaptive neuropsychological functioning was not protective against CT's effect on suicidal risk (19).

Gamma-aminobutyric acid (GABA) is the main inhibitory neurotransmitter in the mammalian brain and, it composes up to one quarter of the total number of neurons in the cortical region. There were increasing amount of studies implied GABA's important role in cognitive deficit in human (20) as well as animal studies (21). Supplementation of GABA showed an acute effect on temporal attention in young healthy adults (22). GABA activity in the hippocampus synchronized the hippocampal-prefrontal cortex pathway which was found to command top-down attention (21).

In our previous studies, we found low expression of a *GABRG2* (GABA A receptor subunit gamma2) isoform which has an alternatively spliced exon in suicide brain (1), then we found that allele A of rs211034, which was located in an intron near the alternatively spliced exon of the *GABRG2* gene, was protective for suicide attempt (SA) (23), these results suggested *GABRG2* as a potential genetic factor in suicide, and rs211034 as a potential site in the risk of suicide. Thus, we hypothesized that this specific genetic variation (rs211034) may interact with reported CT to influence cognition related to suicide or depression risk. The present study aimed to evaluate this hypothesis. This study may provide new evidence for cognitive deficit of suicide and depression, and find the relationship between *GABRG2* and cognitive deficit.

## Methods

### Participants

Two hundred and one adults (Han Chinese, aged from 18 to 65 years old) were recruited from the Department of Psychiatry at Nanfang Hospital (Guangzhou China). Among them, healthy volunteer group was recruited by advertisement. All subjects were interviewed by experienced clinical psychiatrists, and the demographic data including gender, age, marital status, education level, etc and a history of CT and suicidality were recorded. We used the Chinese-Bilingual Structured Clinical Interview for the Diagnostic and Statistical Manual of Mental Disorders, 4th Edition (Axis I, Patient version) (CB-SCID-I/P) to ascertain the diagnosis of any psychiatric disorder for patients and the Non-patient Edition of CB-SCID-I (CB-SCID-I/NP) to evaluate the psychiatric diagnoses, personality disorders and suicidal behaviors for healthy controls. The samples of this study included a small part of samples in the previous study who were diagnosed with MDD and administered with Stroop task, but most of the samples were recruited specifically for the present study because the majority of the samples in the previous study were not administered with Stroop task.

Subjects who met the following criteria were excluded: (a) a history of neurological disease and the presence of psychiatric disorders on either Axis I (e.g., schizophrenia spectrum) or Axis II (personality disorders, mental retardation, etc.), (b) co-morbid substance use disorders, and (c) lacking capacity to provide informed consent. We also excluded participants who have been using mood stabilizers, antidepressants, anxiolytics, antipsychotic and benzodiazepines within the previous 2 weeks in case medications result in potential confounding effects. Healthy controls were evaluated by psychiatrists as regards psychiatric diagnoses, personality disorders and suicidal behaviors using the Non-patient Edition of CB-SCID-I (CB-SCIDI/NP). The controls were excluded if they had: (i) an AxisI or II diagnosis, or (ii) a personal history of suicidal thoughts or attempted suicide.

Fifty two suicide attempters with MDD [depressed suicide attempter (DSA), 12 men, 23.1% and 40 women, 76.9%] and 59 non-attempters with MDD [depressed controls (DC), 24 men, 40.7% and 35 women, 59.3%], and 90 healthy controls (HC, 52 men, 57.8% and 38 women, 42.2%) were enrolled in the study. There were differences in sex ratio and age among the three groups: the control group had fewer females and older subjects compared with mood disorder groups (Table 1).

**Table 1:** Demographic and clinical features, as well as comparison results without adjusting covariates.

**Items**		**DSA** **(***N*** = 52)**	**DNA** **(***N*** = 59)**	**HC** **(***N*** = 90)**	***F*****/χ^2^** **(***p***-value)**	**Significant direction**
		**Mean (sd)/*****N*** **(%)**	**Mean (sd)/*****N*** **(%)**	**Mean (sd)/*****N*** **(%)**		
Age		25.56 (8.53)	30.14 (11.44)	35.19 (9.44)	16.24 (<0.001)	DSA < DNA < HC
Gender	Male	13 (25.0%)	24 (40.7%)	52 (57.8%)	14.79 (<0.001)	Male: DSA < HC
	Female	39 (75.0%)	35 (59.3%)	38 (42.2%)		
Education level high school and above	Yes	38 (73.1%)	39 (66.1%)	48 (53.3%)	6.01 (0.050)	
	No	14 (26.9%)	20 (33.9%)	42 (46.7%)		
Marital status	Single	37 (71.2%)	31 (52.5%)	27 (30%)	26.46 (<0.001)	Single: HC < DNA < DSA
	Married	13 (25%)	28 (47.5%)	61 (67.8%)		
	Divorced	2 (3.8%)	0 (0%)	2 (2.2%)		
Positive words RT		689.09 (251.9)	642.34 (213.89)	572.99 (164.85)	5.57 (0.004)	HC < DNA, DSA
Negative words RT		708.98 (256.56)	646.51 (228.41)	571.41 (160.21)	7.31 (0.001)	HC < DNA, DSA
Suicide words RT		706.46 (254.94)	651.21 (228.82)	581.69 (165.67)	5.83 (0.003)	HC < DNA, DSA
Neutral words RT		702.85 (243.35)	635.87 (213.52)	577.45 (166.79)	6.3 (0.002)	HC < DSA
Positive words IF		−13.75 (59.35)	6.46 (40.46)	−4.45 (43.3)	2.53 (0.083)	
Negative words IF		6.14 (59.43)	10.63 (55.29)	−6.03 (32.67)	2.38 (0.096)	
Suicide words IF		3.61 (47.33)	15.34 (64.35)	3.12 (41.05)	1.15 (0.318)	
Positive words missing number		1.46 (3.15)	0.98 (2.2)	0.3 (0.55)	5.67 (0.004)	HC < DNA, DSA
Negative words missing number		1.35 (2.78)	1.16 (2.41)	0.34 (0.64)	5.25 (0.006)	HC < DNA, DSA
Suicide words missing number		1.46 (3.17)	1.05 (2.7)	0.35 (0.68)	4.25 (0.016)	HC < DSA
Neutral words missing number		1.13 (2.87)	0.86 (2.05)	0.3 (0.63)	3.61 (0.029)	HC < DNA, DSA
rs211034	AA and AG	21 (40.4%)	35 (59.3%)	45 (50%)	5.31 (0.070)	
	GG	31 (59.6%)	21 (35.6%)	40 (44.4%)		
CTQ Sexual Abuse	Yes	14 (26.9%)	8 (13.6%)	7 (7.8%)	9.84 (0.007)	Yes: HC < DSA
	No	38 (73.1%)	51 (86.4%)	83 (92.2%)		
CTQ Physical Abuse	Yes	17 (32.7%)	13 (22%)	4 (4.4%)	20.27 (<0.001)	Yes: HC < DNA, DSA
	No	35 (67.3%)	46 (78%)	86 (95.6%)		
CTQ Emotional Abuse	Yes	20 (38.5%)	13 (22%)	2 (2.2%)	31.34 (<0.001)	Yes: HC < DNA, DSA
	No	32 (61.5%)	46 (78%)	88 (97.8%)		
CTQ Emotional Neglect	Yes	27 (51.9%)	20 (33.9%)	4 (4.4%)	42.44 (<0.001)	Yes: HC < DNA, DSA
	No	25 (48.1%)	39 (66.1%)	86 (95.6%)		
CTQ Physical Neglect	Yes	32 (61.5%)	25 (42.4%)	18 (20%)	25.23 (<0.001)	Yes: HC < DNA, DSA
	No	20 (38.5%)	34 (57.6%)	72 (80%)		
Total CTQ score		67.94 (26.27)	46.49 (20.63)	30.13 (9.54)	69 (<0.001)	HC < DNA < DSA
Suicide attempt recency	Within 1 week	9 (17.3%)	\	\	\	\
	Not within 1 week	42 (80.8%)				
HAMD-24 score		36.21 (6.24)	28.92 (6.91)	\	*t*-stats 5.84 (<0.001)	DNA < DSA

*DSA, depressed suicide attempter; DNA, depressed non-attempter; HC, healthy control; RT, reaction time; IF, interference index; SA/PA, Sexual Abuse/Physical Abuse; EA/EN/PN, Emotional Abuse/Emotional Neglect/Physical Neglect; CTQ, childhood trauma questionnaire*.

Each participant signed a written form of informed consent, approved by the Southern Medical University Clinical Research Ethics Committee (Reference Number: NFEC-2018-041), and the whole process of this study was executed in accordance with guidelines and regulations of the Committee.

## Instruments

### CT Exposure

CT was assessed using the Childhood Trauma Questionnaire Short Form [CTQ-SF; (24)]. It is a self-report measure that serves to retrospectively assess the extent of traumatic experiences in the respondent's childhood (before age 12 years). The five subscales of it include: *Emotional Abuse* (EA), *Physical Abuse* (PA), *Sexual Abuse* (SA), *Physical Neglect* (PN) and *Emotional Neglect* (EN). The CTQ is comprised of 28 items and is a 5-point Likert scale, ranging from 1 (“never true”) to 5 (“very often true”). Levels for each subscale were considered clinically significant within the “moderate to extreme” range, thus there were thresholds for each subscales. The participant who scores higher than 12 in subscale of emotional abuse, 9 in subscale of PA, 7 in subscale of sexual abuse, 14 in subscale of emotional neglect, and 9 in subscale of physical neglect, will be classified as “Yes” in these domains, and “No” if the participant endorsed lower levels (24). Therefore, the samples were divided as non-abused vs. abused or non-neglected vs. neglected group according to the classification of CTQ scores.

### SST

In the current study, E-Prime 2.0 Professional SP1 (2.0.10.356) software was used to present the stimuli for the task and record the response latencies. At the beginning of the task, directions presented on the screen instructed participants to identify the color of each presented word as quickly and as accurately as they can. Each trial started with a blank, white screen for 4 s, then a centered “+” for 1 s. The “+” was followed by a blank screen for 1 s, then replaced by the word printed in red or blue, which remained on the screen until a response was recorded. Participants should identify the color of the words as quickly and as accurately as they can by pressing the F (red) or J (blue) key on the computer keyboard. All the words appeared in the center of the computer screen (17 inches), with black background and the sizes were “song type” 40. The whole experiment was divided into two parts: practice part and experiment part. The words in the practice part are the names of fruits in different colors, and there were 6 trials in total. The experiment part was divided into two blocks, each block contained 12 words of four types. Each word appeared twice and randomly, there was a total of 96 trials in the experiment part.

Twelve positive, 12 negative and 12 neutral words were selected from Chinese Affective Words System (CAWS) (25). Univariate analysis of variance showed that there were significant differences in valence among the three types of words and between each two types (all *p*-values < 0.001); there were significant differences in arousal among the three types of words, although no significant difference in arousal between the positive and negative words (*p*-value = 0.259), the arousal of these two types of words were significantly higher than that of neutral words (all *p*-values < 0.001) (see Table 2). Twelve suicide-related words were selected based on previous studies (7), as well as based on general relevance to suicide. Each category was presented 24 times throughout the task.

**Table 2:** Univariate analysis of variance about the Valence and Arousal of the positive, neutral and negative words selected from Chinese Affective Words System (CAWS).

**Characteristics of words**	**Positive words** **(***N*** = 12)**	**Neutral words** **(***N*** = 12)**	**Negative words** **(***N*** = 12)**	***F***	***p*** **-value**
Valence	7.42 (0.15)	4.96 (0.05)	2.73 (0.17)	3,716.545	<0.001
Arousal	5.25 (0.77)	4.47 (0.52)	5.53 (0.48)	10.001	<0.001

Trials with incorrect responses and with RT ± 2 SD from each participant's mean RT were eliminated. Stroop interference score (IF) was calculated based on the RT. We calculated four IFs for each participant by subtracting RTs for neutral words from RTs for suicide-related words, negative words and positive words. Higher IFs indicate relatively slower response to colors, therefore representing fixation on word content. Missing number for each kind of words was also included in analysis.

### Suicide Attempt

A lifetime history of suicide attempt was ascertained using a semi-structured clinical interview with questions about the medical severity of the attempts and underlying suicidal intent. A suicide attempt was defined as a self-damaging act with intent to die. Patients who exhibited other self-destructive behaviors (e.g., self-mutilation) or suicide ideation without any attempt were not included (26). A suicide attempt can result in injury or at least involve potential for injury. We can determine the suicidal intent through inquiring the individual's intent for the behavior. When determination of the suicidal intent was not possible (e.g., the individual refuses to provide relevant information), suicidal intent can be inferred based on the individual's perception of lethality of the behavior, or an impressive circumstance that leave no doubt regarding other intents (27).

### Severity of Depression

The Hamilton Depression Scale-24 items (HAMD-24) (28) is a widely used rating scale to measure the severity of depressive symptoms. It comprises 24 items, and is based on the clinician's interview with the patient. It detects symptoms such as depressive mood, anxiety, guilty feelings, sleep disturbances, suicide, hopelessness and so on.

### Genotyping

rs211034 (5:162102714) was found to be related with suicidal behavior in our previous research (23). It was located in intron, and 40bp downstream of the alternatively spliced exon of the *GABRG2* gene. Variants located in this area may affect the transcription, splicing, stability and translation of the nearby exon (29). Although rs211035 (5:162102565) was also related with suicidal behavior according to our previous research, it was not genotyped in the present research due to its high linkage disequilibrium with rs211034 (D' = 1.0, *r*^2^ = 0.698).

DNA was extracted from the peripheral blood mononuclear cell (PBMC) fraction using the total DNA isolation kits (Tiangen, Beijing, China). The rs211034 polymorphism was characterized using a standard polymerase chain reaction (PCR). PCR was carried out in a 20-μL reaction mixture comprising 3.0 mM Mg2+, 0.3 mM dNTP, 1× HotStarTaq buffer, 1U HotStarTaq polymerase and 1 μL pure DNA sample. Amplification process was executed as the following 8 steps: 1, initial denaturation at 95°C for 2 min, 2, 11 cycles of denaturation at 94°C for 20 s, 3, annealing at 59.5°C for 40 s, 4, extension at 72°C for 90 s, 5, 24 cycles of denaturation at 94°C for 20 s, 6, annealing at 59°C for 30 s, 7, extension at 72°C for 90 s, 8, final extension at 72°C for 120 s. Primer sequences were forward: 5′-TCCTGGACTTGGTGGATTTCT-3′ and reverse: 5′-TCCCACATAGTTCCCCCTTTC-3′. PCR products were then sent to the Sangon Biotech Company (Shanghai, China) and genotyped using Sanger sequencing (30).

## Statistical Analyses

### Descriptive Analyses and Correlation Analyses

**Figure 1:**
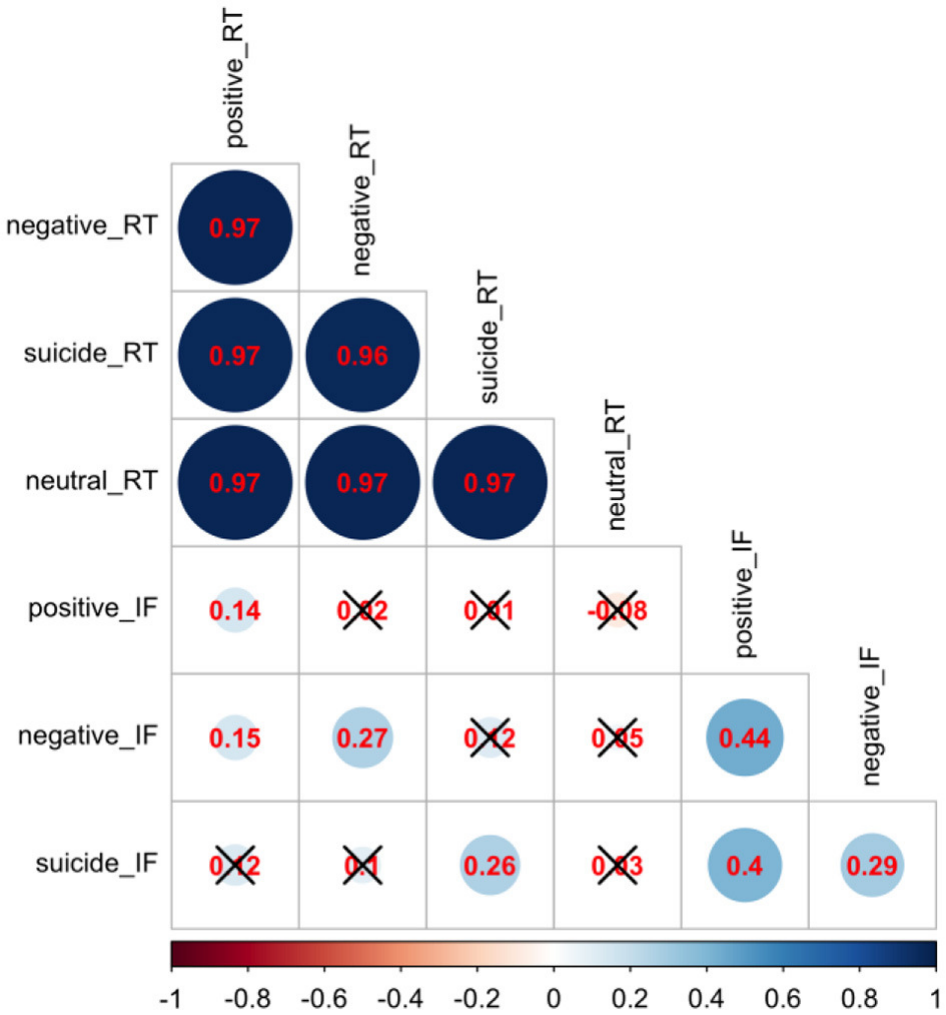
Correlation analysis of Stroop task reaction time and interference scores. Red numbers are pairwise correlations, and a black cross represents an insignificant *p*-value.

**Figure 2:**
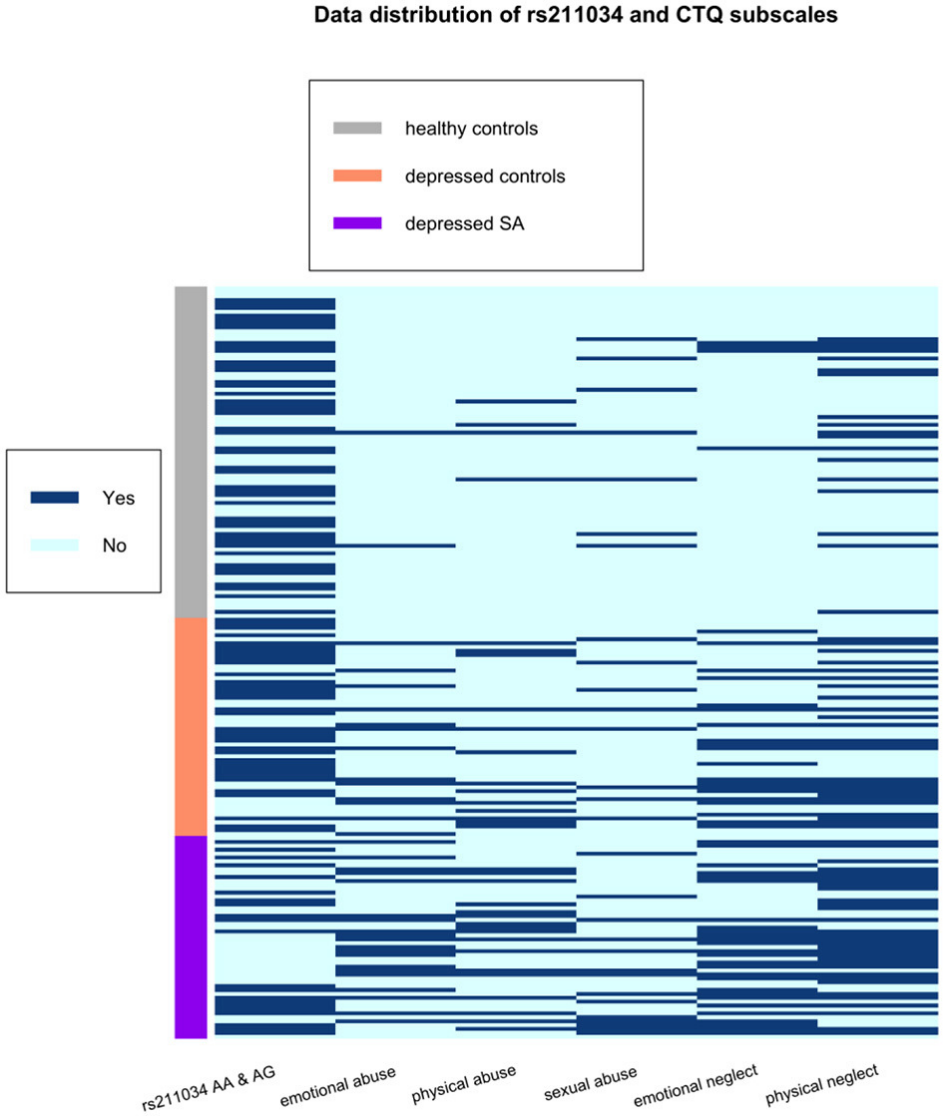
Data distribution of the SNP rs211034 and five CTQ subscales, grouped by patients' suicide attempt status.

All statistical analyses were conducted in R version 4.0.2 (2020-06-22) — “Taking Off Again,” Copyright (C) 2020 The R Foundation for Statistical Computing (31). Descriptive variables of the participants, such as sample proportion, sample mean and sample standard deviation, were calculated by group and showed in Table 1. For univariate analysis without adjusting for other variables, ANOVA F-test and Pearson's chi-squared test were used to compare continuous data and categorical data, respectively, among three groups: healthy controls, depressed non-attempters, and depressed suicide attempters (Table 1). We conducted a correlation analysis of RT and interference effect using Pearson correlation and showed the results in Figure 1. In addition, a heatmap in Figure 2 depicted the data distribution of the SNP rs211034 and five CTQ subscales.

### Association Between Depression/Suicide Attempts and Performance of SST

RT and interference (IF) scores of Stroop Task were compared in three groups: healthy controls, depressed non-attempters, and depressed suicide attempters, using linear regression models. Categorized age, gender (male/female) and education (high school and above vs. others) were treated as covariates in the regression models, where age is categorized to three groups, age of 18–25, age of 26–35, and age of 36–62. Specifically, linear regression models comparing RT and interference (IF) among three groups were conducted, and we highlighted the comparison between healthy controls and depressed non-attempters, and the comparison between depressed non-attempters and depressed suicide attempters. Due to the limited sample size, we conducted a power analysis for the linear regression model of comparing positive word related RT among three groups. Based on (32) and the R-package “pwr” (33), we used *f* ^2^ value (ratio between the additional variance explained by the group variable and unexplained variance) as the effect size. We reported the calculated power under different sample sizes and different effect sizes.

### Interaction Effect Between SNP and CTQ on Performance of SST

We first looked at the association between the SNP rs211034 and performance of SST using regression models, and then included CTQ subscales to explore the interaction effect. Five CTQ subscales (*Physical Abuse, Sexual Abuse, Physical Neglect, Emotional Neglect* and *Emotional Abuse*) were analyzed separately. For each CTQ subscale, three linear regression models were fit in R to examine the association between SNP/CTQ and Stroop Task RT related to positive, negative and suicide words, respectively, adjusting for age group, gender and education level. An interaction term between SNP and CTQ was also included in the models, in order to examine if the genetic effect on RT was different between subjects exposed to reported CT and subjects not exposed to reported CT. We tested the interaction between gender and SNP rs211034, as well as the interactions between gender and the five CTQ subscales, but none of them was significant, so we only included the gender as one of the covariates.

Similar association analyses were conducted for IFs. The interaction effect between SNP rs211034 and each CTQ subscale was of main interest. As there were five CTQ subscales, we used Bonferroni correction for multiple comparisons. Hence, the result would be interpreted as significant when *p*-value was <0.01. If interactions were found, we would look at the main effect of SNP rs211034 within samples exposed to CTQ and samples not exposed to CTQ separately.

## Results

### Descriptive Analyses and Correlation Analyses

Descriptive variables and comparison analyses among depressed suicide attempters, depressed non-attempters, and healthy controls are shown in Table 1. We observed that age is significantly different among depressed suicide attempters (25.56 ± 8.53), depressed non-attempters (30.14 ± 11.44), and healthy controls (35.19 ± 9.44), and that there are significantly more male in healthy controls (52/90, 57.8%) than in depressed suicide attempters (13/52, 25.0%), so we included both age and gender as covariates in the following analyses. There are also some significant results for RT and interference effect, but these results were considered descriptive and were not adjusted for other covariates.

We did a correlation analysis of RT and interference effect and showed the results in Figure 1. It was expected that RT related to different words are highly correlated with each other (*r* > 0.95), as the RT is usually associated with subjects' personality. Interference effect related to different words are also moderately correlated with each other. We also showed the data distribution of the SNP rs211034 and five CTQ subscales using a heatmap in Figure 2, grouped by patients' suicide attempt status.

### Association Between Depression/Suicide Attempts and Performance of SST

**Figure 3:**
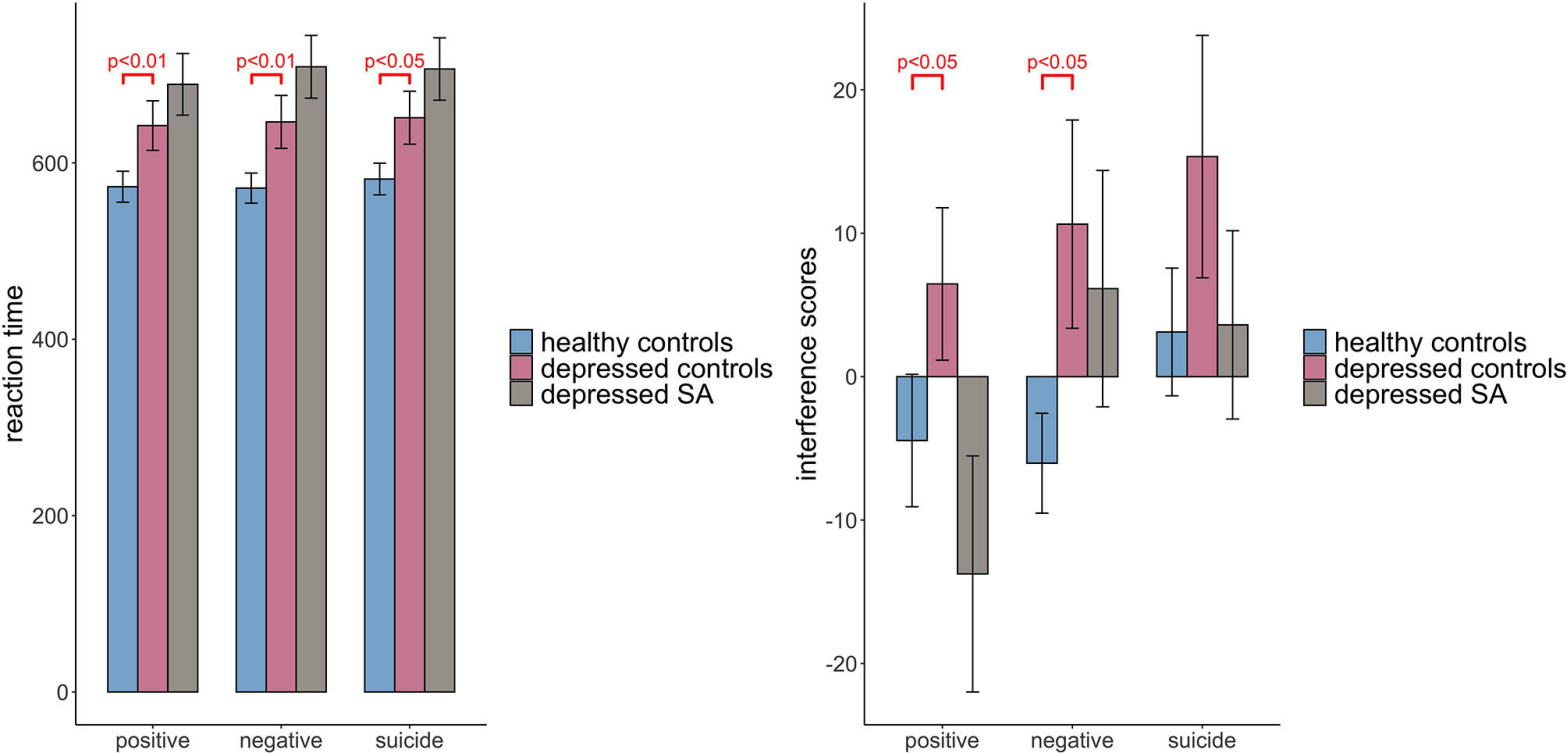
Comparison of Stroop task reaction time and interference scores among three groups: healthy controls, depressed controls and depressed suicide attempters. Raw scores before covariate adjustment are presented, and error bars represent standard error of the mean. Significant results are obtained from regression models adjusting for age, gender and education level.

Table 3 showed the results of linear regression models comparing RT and IFs among healthy controls, depressed non-attempters (reference group) and depressed suicide attempters, adjusting for age group, gender, and education level. It showed that the RT of healthy controls were shorter than that of depressed non-attempters (β = −98.833, *p* = 0.006), for positive words (β = −97.849, *p* = 0.008), for negative words, and (β = −93.795, *p* = 0.012) for suicide words. When comparing depressed suicide attempters and depressed non-attempters, there is no significant difference of RT for positive words (β = 57.383, *p* = 0.135), negative words (β = 69.649, *p* = 0.079), and suicide words (β = 63.622, *p* = 0.112). For IFs, healthy controls showed significantly less positive words interference effect than depressed non-attempters (β = −18.655, *p* = 0.028) and less negative words interference effect (β = −17.670, *p* = 0.043), while they did not differ for suicide words (β = −14.846, *p* = 0.108). When compared depressed suicide attempters and depressed non-attempters, IFs did not differ (β = −16.048, *p* = 0.080), for positive words (β = −3.782, *p* = 0.688), for negative words, and (β = −9.805, *p* = 0.324) for suicide words. Figure 3 showed the mean and standard error of the mean for RT and IFs within healthy controls, depressed controls, and suicide attempters separately, as well as the association results from regression models.

**Table 3:** Parameter estimates in the linear regression models comparing reaction time and interference scores among healthy controls, depressed non-attempters (reference group) and depressed suicide attempters, adjusting for age group, gender, and education level.

**Independent variables**	**Dependent variables**					
	**Positive words**		**Negative words**		**Suicide words**	
	**Estimate**	***p*** **-value**	**Estimate**	***p*** **-value**	**Estimate**	***p*** **-value**
**Block 1: reaction time**						
Status healthy controls	−98.833	0.006	−97.849	0.008	−93.795	0.012
Status depressed SA	57.383	0.135	69.649	0.079	63.622	0.112
**Block 2: interference scores**						
Status healthy controls	−18.655	0.028	−17.670	0.043	−14.846	0.108
Status depressed SA	−16.048	0.080	−3.782	0.688	−9.805	0.324

**Figure 4:**
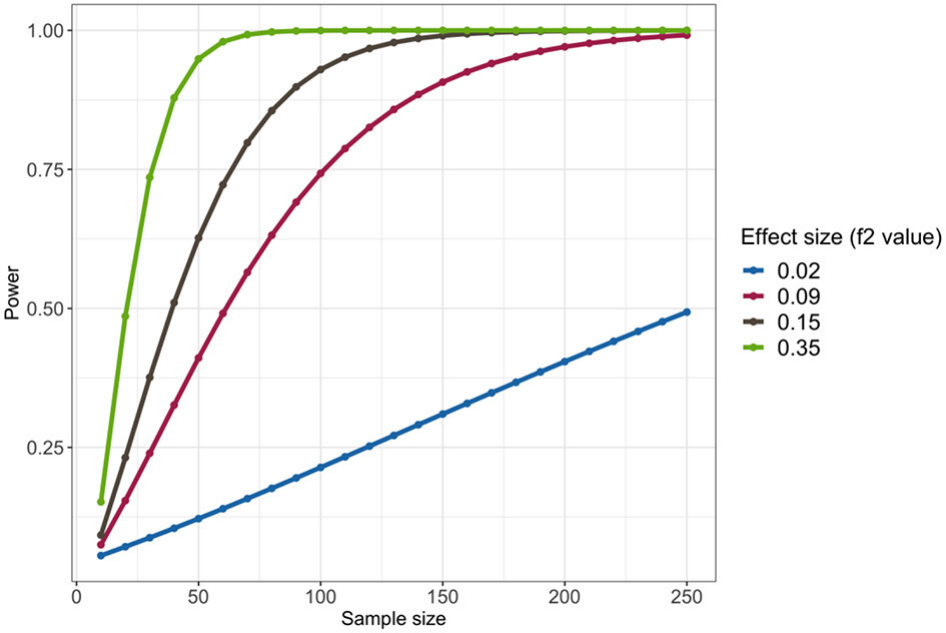
Power analysis of the linear regression model comparing Stroop task reaction time related to positive words among three groups: healthy controls, depressed controls, and depressed suicide attempters, under the significance level 0.05.

We showed the result of power analysis in Figure 4, under the significance level 0.05. In addition to Cohen's suggested *f* ^2^ values of 0.02, 0.15, and 0.35 representing small, medium, and large effect sizes, we also reported the *f* ^2^ values estimated by our model, which was 0.09. It showed that under the small effect size (*f* ^2^ = 0.02), the total sample size of 200 cannot provide a promising power, but when effect size is 0.09 or larger, the total sample size of 200 can provide a power more than 90%. There were totally 201 samples in our data, so we may achieve a good power in this analysis.

### Interaction Effect Between SNP and CTQ on Performance of SST

Results of regression analyses between SNP/CTQ and RT were presented in Table 4, where parameter estimates of different CTQ subscales and interaction with SNP rs211034 were showed in different blocks. Block 0 showed the results of association between SNP and RT without including CTQ. It showed that those with rs211034 allele A had a significant shorter RT time than those without rs211034 allele A (β = −62.471, *p* = 0.039), for positive words (β = −70.994, *p* = 0.020), for negative words, and (β = −66.472, *p* = 0.036) for suicide words (Table 4, Block 0).

**Table 4:** Parameter estimates in the linear regression models testing association between reaction time with SNP rs211034 and different CTQ subscales, and also the interaction, adjusting for age group, gender, and education level.

**Independent variables**	**Dependent variables**					
	**RT related to positive words**		**RT related to negative words**		**RT related to suicide words**	
	**Estimate**	***p*** **-value**	**Estimate**	***p*** **-value**	**Estimate**	***p*** **-value**
**Block 0**						
rs211034: AA and AG	−62.471	0.039	−70.994	0.020	−66.472	0.036
**Block 1**						
rs211034: AA and AG	−92.060	0.004	−103.670	0.001	−97.082	0.004
CTQ Sexual Abuse	−42.140	0.454	−53.812	0.343	−43.997	0.454
CTQ Sexual Abuse X rs211034	202.524	0.014	221.785	0.008	215.121	0.012
**Block 2**						
rs211034: AA and AG	−65.505	0.047	−70.784	0.034	−70.122	0.044
CTQ Physical Abuse	9.761	0.863	13.723	0.810	3.232	0.956
CTQ Physical Abuse X rs211034	10.530	0.894	−9.076	0.909	20.324	0.805
**Block 3**						
rs211034: AA and AG	−69.232	0.038	−75.738	0.025	−77.601	0.027
CTQ Emotional Abuse	−23.932	0.674	0.739	0.990	−31.653	0.594
CTQ Emotional Abuse X rs211034	24.223	0.752	18.771	0.808	54.435	0.496
**Block 4**						
rs211034: AA and AG	−85.663	0.015	−92.841	0.009	−89.065	0.016
CTQ Emotional Neglect	−60.781	0.215	−45.910	0.354	−59.916	0.243
CTQ Emotional Neglect X rs211034	72.732	0.278	71.998	0.288	73.947	0.291
**Block 5**						
rs211034: AA and AG	−56.755	0.144	−64.699	0.098	−52.693	0.199
CTQ Physical Neglect	30.486	0.489	45.791	0.302	43.570	0.346
CTQ Physical Neglect X rs211034	−9.598	0.875	−5.198	0.933	−24.029	0.707

For interaction effect, as we used Bonferroni correction for multiple comparisons, the results of *p*-value < 0.01 would be considered as significant. The interaction effects between CTQ *Sexual Abuse* and SNP rs211034 on reaction were borderline significant (β = 202.524, *p* = 0.014), for positive words (β = 221.785, *p* = 0.008), for negative words, and (β = 215.121, *p* = 0.012) for suicide words (Table 4, Block 1). There was no significant finding for other four CTQ subscales in terms of interaction effect on RT (all *p* > 0.01). For IFs, we didn't observe significant interaction effect for any of the five CTQ subscales.

**Figure 5:**
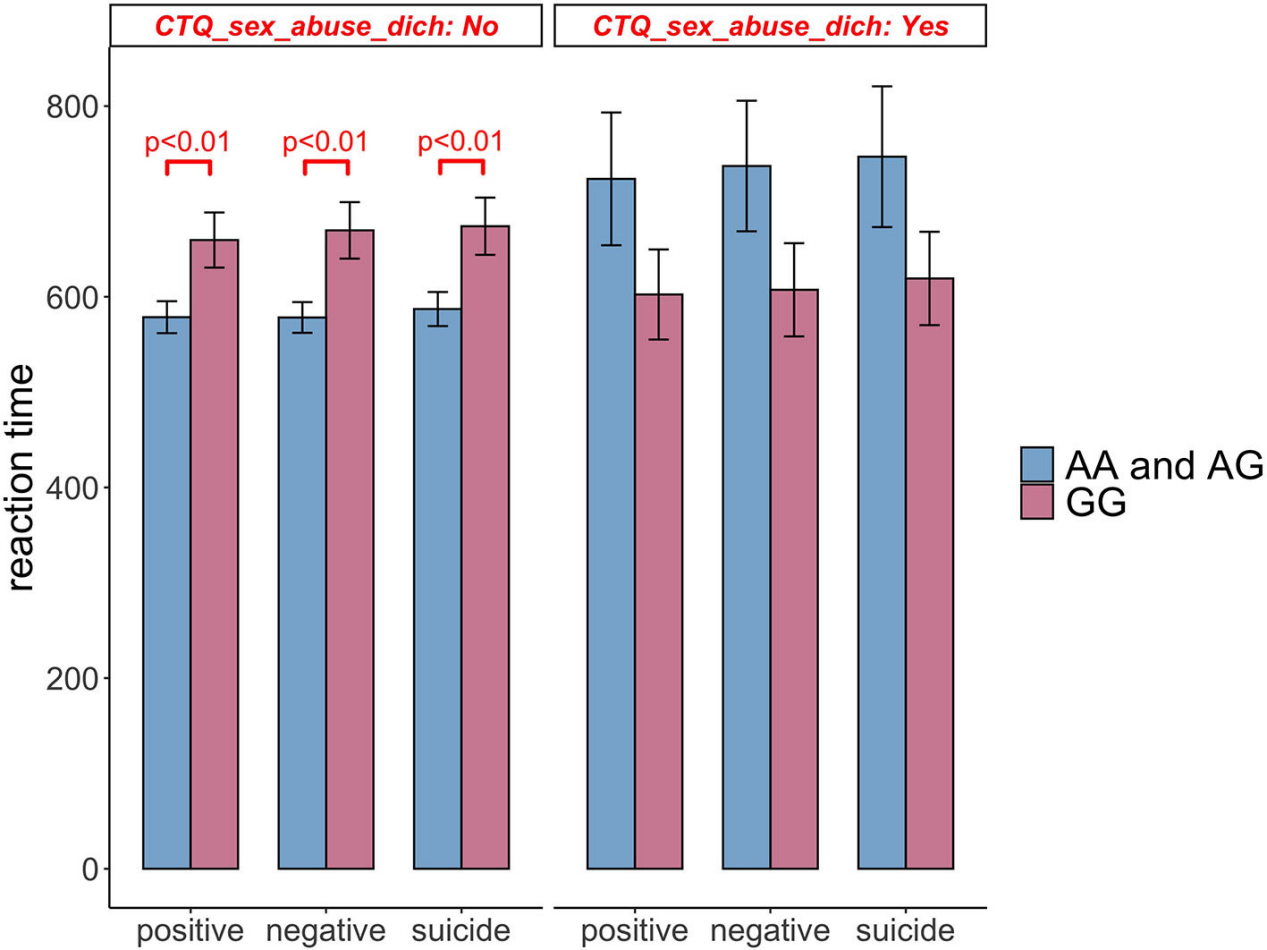
Comparison of Stroop task reaction time between patients having rs211034 allele A and patients not having rs211034 allele A, within the group of subjects exposed to *Sexual Abuse* and the group of subjects not exposed to *Sexual Abuse* separately. Raw scores before covariate adjustment are presented, and error bars represent standard error of the mean. Significant results are obtained from regression models adjusting for age, gender and education level.

In subjects not reporting *Sexual Abuse*, those with rs211034 allele A had significantly shorter RT time than those without rs211034 allele A (β = −92.060, *p* = 0.004), for positive words (β = −103.670, *p* = 0.001), for negative words, and (β = −97.082, *p* = 0.004) for suicide words (Table 4, Block 1). However, in subjects reporting *Sexual Abuse*, those with rs211034 allele A didn't show significant difference in RTs with those without rs211034 allele A (β = 110.464, *p* = 0.143), for positive words (β = 118.115, *p* = 0.121), for negative words, and (β = 118.039, *p* = 0.133) for suicide words. Figure 5 showed the interaction effect by comparing RT between patients having rs211034 allele A and patients not having rs211034 allele A, within the group of subjects not exposed to *Sexual Abuse* and the group of subjects exposed to *Sexual Abuse* separately. Interaction effects for the other four CTQ subscales were not significant.

## Discussion

This study explored the relationship among cognitive performance, *GABRG2* polymorphism rs211034, and CT in depressed attempters/non-attempters. It yielded 3 findings. First, in analyzing suicide-related, negative, and positive word interference on a Suicide-Stroop task, depressed participants had longer RT s for all emotional words and suicide-related words. Second, depressed non-attempters showed higher negative words interference effects than healthy controls, while depressed attempters showed enhanced positive-word response time compared to depressed non-attempters. Third, there was an interaction between rs211034 risk allele A and exposure to *Sexual Abuse*. Specifically, patients who had not experienced prior sexual abuse and carried the rs211034 risk allele A showed shorter RT s for all emotional words and suicide-related words compared with those who did not have this allele, but this difference was not present in patients with reported SA and tended to run in the opposite direction.

We found slowed processing speed in depressed patients, regardless of suicide attempt history. Our findings were consistent with previous studies which consistently find such deficits in depressed patients (34, 35). Processing speed is a factor that may have widespread effects on a number cognitive functions in MDD (35). Previous study observing the neuropsychological function of MDD showed that, patients with treatment-resistant depression exhibited slowed processing speed (36). Moreover, processing speed appears to be associated with response to different treatments. Faster processing speed has been associated with better SSRI response (37), while slower processing speed is a predictor of of positive response to ketamine (38).

Compared to healthy controls, depressed non-attempters had an attention bias to negative words, which was consistent with previous meta-analysis that revealed large depression-related Stroop effects on negative stimuli (39). A recent meta-analysis also concluded that, the MDD groups showed greater interference by negative stimuli (40).

Contrary to our hypotheses, attentional bias to the suicide-related words did not relate to suicide attempt, and this is inconsistent with some previous studies (7, 10), though not all (11, 12). The attentional bias to the suicide-related words may be detectable only among recent suicide attempters, but there were few participants in the present sample (*N* = 9, 17.3%) that had a history of suicide attempt within 1 week of enrollment in the study (see Table 1). Participants who made attempts more than 1 week prior may be less sensitive to suicide related stimuli (41) with RT related to the proximity of the last attempt (12). Attentional bias for suicide-related words was associated only with suicide attempt occurred within the past week in studies taking place in the emergency room (10) or in inpatient units where patients had been admitted for acute clinical care (7).

In the third finding, allele A carriers (rs211034) showed faster processing speed than the individual who didn't carry this allele, which meant that this allele should be a protective factor for processing speed, but when this individual was exposed with childhood sexual abuse (CSA), this protective effect no longer existed. This is consistent with findings examining the modulating effect of childhood abuse on the relationship of neurocognitive deficit to suicidal behavior by Zelazny et al. (19), who found that neurocognitive deficits were related to suicidal behavior outside of the context of abuse, but the relationship tended to be reduced or eliminated when abuse was present. Murphy et al. also found allelic variability in candidate genes may be relevant to suicidal behavior, and a putative gene-environment interaction in suicide risk (42).

rs211034 is located in an intron, and only 40bp downstream of the alternatively spliced exon of the *GABRG2* gene. Variants located in this area may affect the transcription, splicing, stability and translation of the nearby exon (29). This alternatively spliced exon of the *GABRG2* gene is one of the important factors that can influence the function of GABAA receptor as well as, potentially, the risk of suicide. An amino acid fragment, which is encoded by this alternatively spliced exon, inserts into the N-terminal extracellular binding domain of γ 2XL, the longest isoform of the γ 2 subunit of GABAA receptor (*GABRG2*) with a length of 515aa, and inhibits the function of GABAA receptor by preventing its localization on the cell surface (43, 44). Our previous study found decreased prefrontal expression of γ 2XL mRNA *GABRG2*-003 (NM_198903.2) in suicide decedents, suggesting that decreased splicing of this alternatively spliced exon might be a risk factor for suicide (45).

Therefore, based on these previous findings, one speculative explanation for the gene × environment interaction in our study might be that if rs211034 (or a tightly linked SNP) is indeed implicated in splicing of this alternatively spliced exon and thus might influence γ 2XL expression, carriers of the variant allele might be more sensitive to early life sexual abuse and subsequent vulnerability to slowed processing speed. Due to the limited sample size and loss of power stemming from categorization of variables, as well as the observed significant effect of rs211034 in the whole sample without subtyped by environment, we did not expect strong effects of G × E interactions. However, the significant effect of rs211034 in the whole sample was very likely to be driven by the large proportion of samples not being exposed to CT (e.g., only 29 subjects were exposed to Sexual Abuse), and our result was supported by several previous studies. One animal study found that, blockade of prefrontal cortex GABA (A) receptors did increase response latencies, resembling speed of processing deficits (46). There were several studies found that, the function of GABAA receptors could be influenced by some specific gene x CT interactions in the mouse model of early life adversity (47, 48). Growing evidence suggests that epigenetic changes (including DNA methylation, histone modification and so on) are key mechanisms by which CT interact with the genetic factors leading to steady changes in DNA structure, gene expression, cognitive deficits and behavior (49).

We reported unique interaction effect for rs211034 and CSA, but not PA, on processing speed, indicating their possible different influences in neuro-cognition and psychopathology. Previous study demonstrated that, CSA was associated with irregularities in the cortical and subcortical regions of the brain which have been considered to contribute to various cognitive deficits in later life (50). Long-term effect of CSA tends to be larger than the long-term effect of PA, and that CSA may contribute to more long-term problems than PA (51). A recent meta-analysis found that sexual abuse at younger ages was a particularly potent predictor of later suicidal behavior (52). While it is unclear why CSA is a stronger predictor of mental health outcomes, CSA may have greater impact on mental health outcomes due to the re-victimization of sexual abuse victims (53), and offspring exposed to CSA, but not PA, were at greater risk of developing cannabis abuse/dependence compared to those who had not experienced CSA (54).

To our knowledge, the present study is the first to explore the effect of *GABRG2* polymorphism and CSA on cognitive deficit while adjusting for modulating factors in depressed attempters, depressed non-attempters and health controls. Our results suggest a relationship between slower processing speed and depression, and an interaction effect between rs211034 and exposure to CSA on slowed processing speed. The present study also implied role of *GABRG2* in processing speed. Therefore, this work suggests that future research on *GABRG2* might help identify specific groups of depression who might benefit the most from intensive treatment targeting cognitive symptoms or neurocognitive rehabilitation.

## Limitations

There are some limitations of our study that merit discussion. First, the CTQ-SF is a retrospective measure and therefore susceptible to recall biases, although its reliability and validity have been confirmed in clinical and community samples (24). Longitudinal measures are preferable in CT studies, for example a study suggests that the gene × CT interaction might be moderated across development by other environmental factors (55). Second, the age at time of the traumatic experiences was not assessed. Early aversive experiences are presumably associated with internalizing problems, whereas trauma in later childhood is linked to externalizing problems in adolescence and adulthood. Third, limited sample size is a problem. Gene studies require large samples in order to detect G × E interactions, so we did not expect strong effects of G × E interactions in the present study. Fourth, we only included one candidate gene and hence cannot rule out the possibility that there are other genotypes or haplotypes that are directly or indirectly related to attention deficit. Fifth, based on the result that there is significant interaction between SNP rs211034 and CT, it would be better to independently conduct mediation analysis on different subgroups stratified by CT. However, due to the small sample size of subjects being exposed to CT in our sample, we decided not to conduct mediation analysis. We would recruit more subjects for mediation analysis in future study. Finally, other types of genetic variation (i.e., copy number variation) as well as epigenetic markers including DNA methylation should be examined in future studies.

## Conclusions

In summary, our study provides first insight into the relationship between *GABRG2* and cognitive deficit, suggesting that polymorphism of the *GABRG2* gene can interact with CSA to influence the processing speed, and that slowed processing speed may be a mediator of the association between gene-CSA interaction and depression.

## Data Availability Statement

The data that support the findings of this study are available from the corresponding author upon reasonable request.

## Ethics Statement

The studies involving human participants were reviewed and approved by Southern Medical University Clinical Research Ethics Committee. The patients/participants provided their written informed consent to participate in this study.

## Author Contributions

HY was responsible for the conception, design and execution of the reported study, and the writing of the manuscript. JG was responsible for the statistical analysis of the study and helped with the writing of the manuscript. HY, XX, and EL were responsible of the recruitment of participants including interviewing and the psychiatric scales. HY and QX were responsible for the Stroop task. HY, QX, and SZ were responsible for the blood sampling, DNA extraction and PCR. JK helped with the execution of the Stroop task, interpretation of the results and edit of the manuscript. JM helped with the design of the study and edit of the manuscript. All authors contributed to the article and approved the submitted version.

## Funding

This research was funded by National Natural Science Foundation of China (NSFC) (Grant number: 81801351, PI: HY).

## Conflict of Interest

The authors declare that the research was conducted in the absence of any commercial or financial relationships that could be construed as a potential conflict of interest.

## Publisher's Note

All claims expressed in this article are solely those of the authors and do not necessarily represent those of their affiliated organizations, or those of the publisher, the editors and the reviewers. Any product that may be evaluated in this article, or claim that may be made by its manufacturer, is not guaranteed or endorsed by the publisher.
